# Immune Checkpoint Inhibitors Regulate K^+^ Channel Activity in Cytotoxic T Lymphocytes of Head and Neck Cancer Patients

**DOI:** 10.3389/fphar.2021.742862

**Published:** 2021-08-27

**Authors:** Vaibhavkumar S. Gawali, Ameet A. Chimote, Hannah S. Newton, Manuel G. Feria-Garzón, Martina Chirra, Edith M. Janssen, Trisha M. Wise-Draper, Laura Conforti

**Affiliations:** ^1^ Department of Internal Medicine, Division of Nephrology, University of Cincinnati, Cincinnati, OH, United States; ^2^ Division of Immunobiology, Cincinnati Children’s Hospital Medical Center, Cincinnati, OH, United States; ^3^ Immunology, Janssen Research and Development, Spring House, PA, United States; ^4^ Department of Internal Medicine, Division of Hematology Oncology, University of Cincinnati, Cincinnati, OH, United States

**Keywords:** ion channels, immune checkpoint inhibitors, KCa3.1 (intermediate-conductance Ca^2+^-activated K^+^ channel), Kv1.3 channel, Ca^2+^ signalling, head and neck (H&N) cancer, immunotharapy

## Abstract

Programmed death receptor-1 (PD-1) and its ligand (PD-L1) interaction negatively regulates T cell function in head and neck squamous cell carcinoma (HNSCC). Overexpression of PD-1 reduces intracellular Ca^2+^ fluxes, and thereby T cell effector functions. In HNSCC patients, PD-1 blockade increases KCa3.1 and Kv1.3 activity along with Ca^2+^ signaling and mobility in CD8^+^ peripheral blood T cells (PBTs). The mechanism by which PD-L1/PD-1 interaction regulates ion channel function is not known. We investigated the effects of blocking PD-1 and PD-L1 on ion channel functions and intracellular Ca^2+^ signaling in CD8^+^ PBTs of HNSCC patients and healthy donors (HDs) using single-cell electrophysiology and live microscopy. Anti-PD-1 and anti-PD-L1 antibodies increase KCa3.1 and Kv1.3 function in CD8^+^ PBTs of HNSCC patients. Anti-PD-1 treatment increases Ca^2+^ fluxes in a subset of HSNCC patients. In CD8^+^ PBTs of HDs, exposure to PD-L1 reduces KCa3.1 activity and Ca^2+^ signaling, which were restored by anti-PD-1 treatment. The PD-L1-induced inhibition of KCa3.1 channels was rescued by the intracellular application of the PI3 kinase modulator phosphatidylinositol 3-phosphate (PI3P) in patch-clamp experiments. In HNSCC CD8^+^ PBTs, anti-PD-1 treatment did not affect the expression of KCa3.1, Kv1.3, Ca^2+^ release activated Ca^2+^ (CRAC) channels, and markers of cell activation (CD69) and exhaustion (LAG-3 and TIM-3). Our data show that immune checkpoint blockade improves T cell function by increasing KCa3.1 and Kv1.3 channel activity in HNSCC patients.

## Introduction

Head and neck squamous cell cancer (HNSCC) is the seventh most common cancer worldwide (Chow, 2020; Sung et al., 2021). Conventional treatments include surgery, radiotherapy, chemotherapy, and multimodal approaches. However, the prognosis of locally advanced disease remains poor, with a 5 years overall survival <50% (Chow, 2020). The discovery of immunotherapy has changed the landscape of HNSCC treatment by offering long term response in a subset of patients. Programmed cell death receptor-1 (PD-1) immune checkpoint inhibitors have shown promising responses in the treatment of HNSCC. Hence, they have been approved by the FDA since 2016 (Sharma et al., 2017; Chow, 2020). However, over 80% of HNSCC patients do not respond to PD-1 blockade (Bauml et al., 2019). Resistance to immunotherapy remains a big challenge as the majority of patients do not show a response to the treatment (primary resistance) (Sharma et al., 2017). Furthermore, some patients who initially respond and receive benefit from the treatment, later show disease relapse (acquired resistance) (Sharma et al., 2017). Therefore, better understanding of PD-1 signaling is necessary to develop new therapeutic options. The interaction of PD-1 ligand -1 and 2 (PD-L1/2) with their receptor PD-1 on cytotoxic T cells negatively regulates T cell function and causes apoptosis, anergy, and exhaustion (Zhou et al., 2017). PD-L1 is constitutively present in T cells and other immune cells, and it’s over expression by tumor cells contributes to immune evasion (Zhou et al., 2017). PD-L1 is also present, and biologically active, in the plasma of HNSCC patients in a secreted form and bound to exosomes contributing to immune evasion (Theodoraki et al., 2018). However, the mechanisms of PD-L1-mediated PD-1 signaling on T cell function are not fully understood.

Ion channels in T lymphocytes orchestrate the influx of intracellular Ca^2+^ required for downstream effector functions, such as migration and proliferation. Calcium release activated Ca^2+^ (CRAC), voltage-gated K^+^ (Kv1.3) and Ca^2+^ activated K^+^ (KCa3.1) channels are involved in the early phase of T cell activation and regulate the Ca^2+^ influx necessary for their effector functions. Ca^2+^ influx is triggered by the T cell receptor (TCR) mediated-depletion of Ca^2+^ stores in the endoplasmic reticulum and opening of the CRAC channels, and it is aided by Kv1.3 and KCa3.1 channels, which allow maintenance of the negative membrane potential necessary for Ca^2+^ influx through the CRAC channels (Feske et al., 2015). KCa3.1 channels also control chemotaxis (Chimote et al., 2018; Chimote et al., 2020). Defective ion channel function leads to alterations in Ca^2+^ signaling and downstream effector functions (Feske et al., 2006). In HNSCC patients, reduced expression of Kv1.3 in tumor infiltrating lymphocytes (TILs) contributes to lower Ca^2+^ response and cytotoxicity (Chimote et al., 2017). Furthermore, reduced activity of KCa3.1 in CD8^+^ peripheral blood T cells (PBTs) of HNSCC patients causes these cells to be hypersensitive to adenosine found in the tumor microenvironment, ultimately reducing their migratory abilities and restricting their infiltration into tumors (Chimote et al., 2013; Chimote et al., 2018).

It has been reported that overexpression of PD-1 influences TCR-dependent effector functions, such as Ca^2+^ fluxing, secretion of cytokines and cytotoxic activity (Wei et al., 2013). Furthermore, TILs of melanoma patients with high expression of PD-1 have decreased Ca^2+^ responses to TCR stimulation (Chapon et al., 2011). We have recently shown that treatment of HNSCC patients with pembrolizumab, a monoclonal blocking antibody against PD1 (αPD-1), increases KCa3.1 and Kv1.3 activities in CD8^+^ TILs and PBTs, along with their Ca^2+^ fluxing and migratory abilities (Newton et al., 2020). Furthermore, a specific pattern of K^+^ channel reinvigoration was associated with a pathological response to therapy (Newton et al., 2020). However, these studies did not allow us to dissect the mechanisms by which PD-1 signaling affects ion channel activity. Herein, we conducted *in vitro* studies that further our understanding of the interconnection between PD-1 and ion channels in T cells. These studies showed that blockade of PD-L1/PD-1 interaction leads to the rapid activation of KCa3.1 and Kv1.3 channels to ultimately regulate Ca^2+^ signaling in HNSCC patients’ T cells.

## Materials and Methods

### Human Subjects

Peripheral blood samples from de-identified HNSCC patients (*n* = 32) were obtained from the University of Cincinnati Medical Center. HNSCC patients included in this study were treatment-naïve and had a positive diagnosis of HNSCC by tissue biopsy (See Table 1 for a summary of patient demographics and Supplementary Table S1 for clinical information). Peripheral blood samples of 7 healthy donors (HDs, 4 males and 3 females, age range between 30 and 65 years) were collected from individual donors and from discarded blood units (Hoxworth Blood Center, University of Cincinnati). The demographics of the donors from Hoxworth Blood center were not available. Informed consent was obtained from all HNSCC patients and HDs. The data collected in the study were managed using the Research Electronic Data Capture (REDCap) tools licensed to the University of Cincinnati. Sample collection was approved by the University of Cincinnati Institutional Review Board (IRB no. 2014-4755).

**TABLE 1:** Demographic and clinical data of HNSCC patients enrolled in the study.

Age (at the time of sample collection)	Years
Range	18–90
Mean	59
**Variable**	**Number (%)**
**Sex**	
Male	23 (71)
Female	9 (39)
**Site**	
Oral Cavity	10 (31)
Oropharynx	14 (43)
Larynx	07 (21)
Hypopharynx	0 (0)
Nasopharynx	0 (0)
Unknown Primary	1 (3)
**Primary Tumor**	
T1	8 (25)
T2	10 (31)
T3	5 (16)
T4	8 (25)
Unknown	1 (3)
**Nodal Status**	
N0	7 (21)
N1	11 (34)
N2	11 (34)
N3	3 (9)
Unknown	0 (0)
**ECOG performance status**	
0	6 (19)
1	13 (41)
2	4 (12)
3	1 (3)
Unknown	8 (25)
**Smoking**	
No (<10 pack years)	14 (44)
Yes (>10 pack years)	18 (56)
**Alcohol**	
No (<5 drinks/week)	22 (69)
Yes (>5 drinks/week)	9 (28)
Unknown	1(3)
**p16 status**	
Positive	12 (38)
Negative	13 (41)
Unknown	7 (22)

HNSCC patients (*n* = 32) were enrolled in the study upon fulfillment of eligibility criteria. TNM staging system was used to stage tumor size and nodal involvement. T1 to T4 refers to the size and invasion of the tumors. N1 to N3 refers to the assessment of number and location of the regional lymph nodes. The ECOG (Eastern Cooperative Oncology Group) performance status indicates daily quality of life of individuals affected by diseases on a scale of 0–5. Smoking status (pack years) was calculated by multiplying the number of packs of cigarettes smoked per day by the number of years the person has smoked. We used a cutoff of 10 packs per year to differentiate the smoking status.

### Reagents and Chemicals

Human serum, l-glutamine, sodium hydroxide, poly-l-lysine, LY294002 HCl, ionomycin, calmodulin, poly-l-lysine, thapsigargin (TG), tetraethylammonium-chloride (TEA-Cl), 1,2-Bis(2-Aminophenoxy)ethane-*N,N,N′,N′*-tetraacetic acid (BAPTA), MgCl_2_ were purchased from Millipore Sigma. Sterile, 4-(2-hydroxyethyl)-1-piperazineethanesulfonic acid (HEPES), RPMI-1640 medium, fetal bovine serum, penicillin, streptomycin, Fura-2 and phosphate buffered saline (PBS) were obtained from ThermoFisher. Phosphatidylinositol 3-phosphate diC16 (PI3P) was purchased from Echelon Biosciences. Pembrolizumab (Merck Sharpe and Dohme Corp) samples used in this study were received from the leftover pharmacy supply at Cincinnati Children’s Hospital. Atezolizumab was purchased from Biovision life sciences. PD-L1-Fc chimera was obtained from R&D systems. Stock solutions of LY394002 and TG were prepared in dimethyl sulfoxide and used at 0.1% dilution. Stock solutions of PD-L1-Fc, pembrolizumab and atezolizumab were prepared in sterile PBS.

### Cell isolation and *in vitro* Activation

Peripheral blood mononuclear cells were isolated from whole blood by Ficoll-Paque density gradient centrifugation (Cytiva) as previously described (Chimote et al., 2018). CD8^+^ PBTs were isolated by negative selection using EasySep Human CD8^+^ T cell Enrichment kit (StemCell Technologies). Post isolation, CD8^+^ PBTs were maintained in RPMI-1640 medium supplemented with 10% human serum, 200 U/ml penicillin, 200 mg/ml streptomycin, 1 mM l-glutamine, and 10 mM HEPES. Activation of cells was performed either by adding 40.5 nM of phorbol-12-myristate-13-acetate (PMA, Millipore Sigma) and 1.5 µM of ionomycin (Millipore Sigma) or in cell culture plates coated with 10 μg/ml anti-CD3 and anti-CD28 antibodies (BioLegend) for 72–96 h (h) as previously described (Chimote et al., 2016; Chimote et al., 2018). After isolation, some fresh cells were used for functional studies, the remaining cells were frozen and used later on for flow cytometry experiments.

### Treatment With αPD-1/PD-L1 Antibodies and PD-L1-Fc

CD8^+^ PBTs from HNSCC patients were activated for 72–96 h using PMA and ionomycin followed by treatment with the αPD-1 antibody pembrolizumab (10 μg/ml) and/or the αPD-L1 antibody atezolizumab (1 and 10 μg/ml) for 6 h prior to performing functional studies. CD8^+^ PBTs from HDs were plated on cell culture plates coated with PD-L1 (PD-L1-Fc, R&D Systems) (10 μg/ml) and activated with either PMA and ionomycin or (with anti-CD3/CD28 antibodies for 72–96 h followed by treatment with the αPD-1 antibody pembrolizumab (10 μg/ml) for 6 h before performing functional studies. For prolonged treatment with PD-L1, cells were activated for 120 h with plate-bound anti-CD3/CD8 antibodies along with PD-L1-Fc in T cell medium supplemented with 20 IU/ml of IL-2.

### Electrophysiology

Whole cell patch-clamp electrophysiology was used to measure the activity of KCa3.1 and Kv1.3 channels in activated CD8^+^ PBTs cells of HDs and HNSCC patients as described previously (Chimote et al., 2020). The external solution consisted of (in mM): 160 NaCl, 4.5 KCl, 1 MgCl_2_, 10 HEPES, pH 7.4. Internal solution consisted of (in mM): 145 K-Aspartate, 2 MgCl_2_, 8.5 CaCl_2_, 10 EGTA, 10 HEPES, pH 7.2 TRIS buffer (1 μM free Ca^2+^ concentration). Borosilicate glass (World Precision Instruments) pipettes (4–5 MΩ resistance) were fabricated using a P-97 horizontal puller (Sutter Instruments). A voltage-ramp pulse protocol (from −120 to + 50 mV, for 200 ms, holding potential of −70 mV, every 15 s) was used to elicit the currents from CD8^+^ PBTs. Currents were recorded, amplified and digitalized (Axon 200B and Digidata 1320A, Molecular Devices) through a 16-bit A-D/D-A interface. Data acquisition was performed using pClamp 8.0 software (Molecular Devices) and signals were low pass filtered at 2 kHz and digitalized at 100 kHz. The conductance (G) of KCa3.1 channels was calculated as, the ratio of linear fraction of the currents to the slope of ramp voltage stimulus (measured in the voltage range between −100 and −80 mV) after subtraction of leak current. (Chimote et al., 2018). The G of Kv1.3 channels was determined from the same recordings by measuring the peak currents at +50 mV after subtraction of KCa3.1 currents extrapolated by linear regression. This protocol accurately record and separate KCa3.1 and Kv1.3 currents (Newton et al., 2020). LY294002 was delivered extracellular *via* a manual perfusion system. Calmodulin and PI3P were dissolved in the internal solution and delivered intracellularly *via* the patch-pipette**.** The KCa3.1 and Kv1.3 Gs were measured in at least three to five cells for each condition per individual patient.

For divalent free (DVF) currents, cells patched in the whole-cell configuration were pre-incubated with TG (1 µM) in external solution without Ca^2+^ for 10 min followed by perfusion with 20 mM Ca^2+^ for 1 min and, finally, with DVF solution for 2 min (Vaeth et al., 2017). DVF currents were measured to amplify the CRAC currents. The DVF Ringer’s solution contained (in mM): 150 NaCl, 10 HEDTA, 1 EDTA and 10 HEPES (pH 7.4 with NaOH). The 20 mM Ca^2+^ external solution consisted of (in mM): 130 NaCl, 4.5 KCl, 20 CaCl_2_, 10 D-glucose and 5 HEPES (pH 7.4 with NaOH). 10 mM TEA-Cl was added to all extracellular solutions to block voltage-gated K^+^ channels. The pipette solution contained (in mM): 135 Cs aspartate, 8 MgCl_2_, 8 BAPTA and 1 HEPES (pH 7.2 with CsOH). A ramp (-100 to +100 mV, holding potential of +30 mV) protocol was used for 100 m every 1.5 s to record DVF currents. Analysis of DVF current was performed by measuring the peak current value at the voltage of −100 mV during the ramp step protocol.

### Ca^2+^ Flux Measurements

Intracellular Ca^2+^ fluxes were measured in activated CD8^+^ PBTs using the ratiometric Ca^2+^ sensitive dye Fura-2. Perfusion with the sarco-endoplasmic pump inhibitor TG allowed us to measure Ca^2+^ fluxes that are independent of TCR stimulation and only rely on downstream signaling events initiated by the release of Ca^2+^ from the endoplasmic reticulum (Robbins et al., 2005). T cells were plated on poly-l-lysine coated coverslips followed by treatment with 1 µM Fura-2 AM (ThermoFisher) at 37°C for ∼30 min. Cells were then washed with RPMI-1640 and maintained at 37°C prior to recordings using InCyt-Im2 Ca^2+^ imaging system (Intracellular Imaging). Coverslips were mounted on the microscope and perfused with a Ca^2+^ free solution for 5 min, followed by perfusion with 1 μMTG in Ca^2+^ free solution for other 5 min to deplete the Ca^2+^ from the intracellular Ca^2+^ stores and open the CRAC channels. Finally, 0.5 mM Ca^2+^ solution was perfused for 10–15 min to allow Ca^2+^ influx through CRAC channels. Ca^2+^ free solution had the following composition (in mM): 155 NaCl, 4.5 KCl, 1 MgCl_2_, 10 HEPES, 10 glucose, 2 EGTA, pH 7.4. The 0.5 mM Ca^2+^ solution had the following composition (in mM): 155 NaCl, 4.5 KCl, 2.5 MgCl_2_, 10 HEPES, 10 glucose, 0.5 CaCl_2_, pH 7.4. A standard calibration curve was used to correlate ratiometric Fura-2 values (340/380 nm ratio) with known Ca^2+^ concentrations as per the protocol provided by the manufacturer. Changes in Ca^2+^ values [Delta (Δ) Ca^2+^], a measure of Ca^2+^ fluxing ability, were determined as the difference between the peak of Ca^2+^ reached after 0.5 mM Ca^2+^ and the baseline Ca^2+^ after the perfusion with 1 μMTG in Ca^2+^ free solution, immediately before the addition of 0.5 mM Ca^2+^. Statistical analysis was performed to detect significant difference in ΔCa^2+^ values post treatment for an individual patient or healthy donor. Only those donors who showed a statistically significant increase in the Ca^2+^ values before and after αPD-1 were included in the positive response (PR) group while those who did not show any statistically significant increase were included in the no-response (NR) group. Individual single cell ΔCa^2+^ values from donors in the PR group and the NR group were then combined to detect significance differences between the two groups. This is because of low sample quantity and variable cell count per patient.

### Flow Cytometry

CD8^+^ PBT cells were maintained at a density of 1 × 10^6^ cells/mL and stimulated with PMA/ionomycin for 72 h. Cells were then rinsed with 1x PBS, followed by staining for flow cytometry. The proportion of dead cells were determined using the Zombie UV fixable viability kit (Biolegend). The cells were then fixed with 1% paraformaldehyde (ThermoFisher), washed with 1x PBS and stained overnight with mouse anti-human anti-KCa3.1 biotinylated antibodies (clone 6C1, Alomone). Then, the cells were washed with 1x PBS and incubated for 30 min at room temperature with the following anti-human antibodies (all from Biolegend): anti-CD8-PacificBlue (cloneHIT8A), anti-CD69-APCFire750 (clone FN50), anti-LAG3-BV510 (clone 11C3C56), anti-PD1-BV605 (clone EH12. 2H7), anti-TIM3-BV786 (clone F38-2F2), and anti-streptavidin-PECy7 (clone). Next, the cells were washed with 1x PBS, and permeabilized with BD Cytofix/Cytoperm kit (BD Biosciences) as per the manufacturer’s instructions, and incubated for 30 min at 4°C with mouse anti-human anti-Calmodulin-PerCP (clone 2D1, NOVUS Biologicals), and anti-human anti-Ki67-BV711 (Biolegend).

For determination of ion channel expression, cells were fixed with 4% paraformaldehyde, washed with PBS followed by overnight incubation at 4°C with the following primary antibodies, mouse anti-human KCa3.1 (6C1/ATTO-488), guinea pig anti-human Kv1.3, rabbit anti-human Orai1 (all from Alomone labs) followed by anti-guinea pig (Dy350 goat anti-guinea pig IgG/Thermo Fisher) and anti-rabbit (Alexa Fluor 594 goat anti-rabbit IgG/Thermo Fisher) secondary antibodies. To stain for intracellular ion channel epitopes, cells were permeabilized with BD Cytofix/Cytoperm kit (BD Biosciences) as per manufacturer’s instructions. Cells were then stained for rabbit anti-human STIM1 (Proteintech) primary antibodies followed by secondary antibodies (Alexa Fluor 594 goat anti-rabbit IgG/Thermo Fisher). Specificity of these antibodies was previously reported by our laboratory (Chimote et al., 2018; Newton et al., 2020). All flow cytometry data were collected on LSR Fortessa or LSR II flow cytometer (BD Biosciences), using the FACS Diva software v.6.0. At least 30,000 total events were acquired. Fluorescence minus one controls were also included. The flow cytometry data were analyzed using FlowJo Software version 10.6.1 ((BD Biosciences).

### Statistical Analysis

Statistical analyses were performed using Student’s *t* test (paired or unpaired), Mann-Whitney rank sum test (in experiments where samples failed normality or had unequal variance), and ANOVA or ANOVA on Ranks as indicated. Post hoc testing on ANOVA was done by multiple pairwise comparison procedures using the Holm-Sidak, Dunn’s or Tukey’s methods depending on sample normality and variance. Statistical analysis was performed using SigmaPlot 13.0 (Systat Software Inc.). *p* value of less than or equal to 0.05 was considered as statistically significant.

## Results

### PD-1 Blockade improves KCa3.1 and Kv1.3 Activity in CD8^+^ PBTs of HNSCC Patients

**FIGURE 1:**
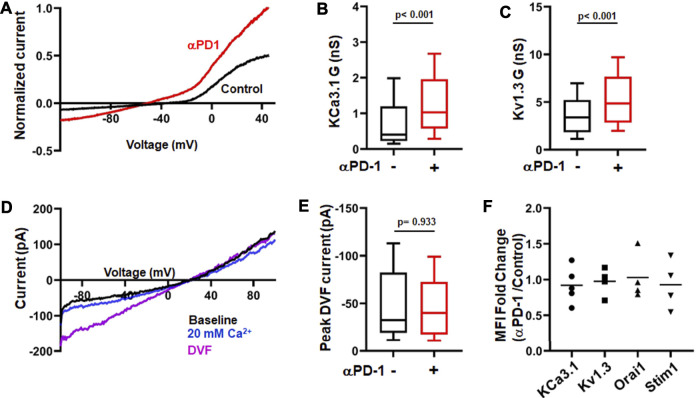
αPD-1 treatment increases K^+^ channel activity in HNSCC T cells. **(A)** Representative current traces of KCa3.1 and Kv1.3 channels recorded in whole-cell mode of voltage-clamp configuration in activated CD8^+^ PBTs cells from a HNSCC patient in absence or presence of αPD-1 (10 μg/ml, for 6 h). Data are normalized to maximum current at +50 mV recorded using a ramp pulse protocol from −120 mV to +50 mV for 200 ms every 15 s. The holding potential used was −70 mV. **(B,C)** KCa3.1 **(B)** and Kv1.3 **(C)** conductance **(G)** measured in the absence or presence of αPD-1 (10 μg/ml, 6 h incubation) in CD8^+^ PBTs of HNSCC patients (*n* = 68 cells without pembrolizumab and *n* = 55 cells with pembrolizumab from 14 patients). **(D)** Representative current traces of divalent free current (DVF) through CRAC channels recorded in whole-cell mode of voltage-clamp configuration in activated CD8^+^ PBTs from a HNSCC patient. Data were recorded using a ramp pulse protocol from −100 to +100 mV with at holding potential of +30 mV every 1.5 s. Cells were perfused with 0 mM Ca^2+^ solution (1 min) followed by 20 mM Ca^2+^ (1 min) and DVF solutions (2 min, see methods) to amplify currents during recordings. **(E)** Peak DVF current values measured in absence and presence of αPD-1 (10 μg/ml, 6 h incubation) in CD8^+^ PBTs of HNSCC patients (*n* = 34 cells without αPD-1 and *n* = 31 cells with αPD-1 from 8 patients). The values in panels **(B,C)** and **(E)** are represented as box plots: the horizontal line indicates the median; the lower box is the 25^th^ percentile; the upper box is the 75^th^ percentile; and the whiskers represent the 10^th^ and 90^th^ percentiles. **(F)** Ion channel expression (KCa3.1, Kv1.3, Orai1 and STIM1) in HNSCC patient T cells after treatment with αPD-1 (10 μg/ml for 6 h). Effect of αPD-1 treatment is shown as ratio of mean fluorescence intensity (MFI, fold change) values of treatment versus control group. Data are represented as scatter plot where each symbol represents an individual patient (*n* = 4–5). Horizontal line represents mean values for each group. Data in panels **(B,C,E)** were analyzed by Mann-Whitney rank sum test.

Ion channels are fundamental regulators of T cell Ca^2+^ signaling and effector functions (Feske et al., 2015) and we have shown that treatment of HNSCC patients with pembrolizumab increases KCa3.1 and Kv1.3 activity in CD8^+^ PBTs (Newton et al., 2020). Herein, we conducted *in vitro* experiments to study in detail the effect of pembrolizumab on ion channels in HNSCC T cells. We tested the effect of a short exposure (6 h) to pembrolizumab (αPD-1 antibody) (10 μg/ml) on activated CD8^+^ PBTs. Whole-cell currents were measured before and after αPD-1 treatment to assess KCa3.1 and Kv1.3 activity (Figure 1A). αPD-1 treatment increased the conductance of both KCa3.1 and Kv1.3 channels (Figures 1B,C). We did not detect any effect of αPD-1 on CRAC channel activity (Figures 1D,E). Further control experiments ruled out the possibility that the increase in KCa3.1 and Kv1.3 activity was an artefact due to the extra 6 h the cells treated with αPD-1 were maintained in culture. The KCa3.1 and Kv1.3 conductance in untreated cells at the beginning of the experiment (0 h) and after 6 h (equivalent to the time of exposure to αPD-1) were comparable (Supplementary Figure S1). There was no difference in the capacitance values, an arbitrary measure of cell size and activation state, before and after treatment with pembrolizumab (Supplementary Figure S2). Furthermore, flow cytometry experiments showed no changes in the expression of either ion channels or activation and exhaustion markers in CD8^+^ PBTs from HNSCC patients after αPD-1 treatment (Figure 1F; Supplementary Figures S3–S4). These findings indicate that αPD-1 treatment increases KCa3.1 and Kv1.3, but not CRAC channel, activity in CD8^+^ PBTs of HNSCC patients. Furthermore, αPD-1 treatment did not change the expression of typical markers associated with exhaustion and activation in CD8^+^ PBTs of HNSCC patients.

### PD-1 Blockade increases Ca^2+^ Fluxing Abilities in T Cells From a Subset of HNSCC Patients

**FIGURE 2:**
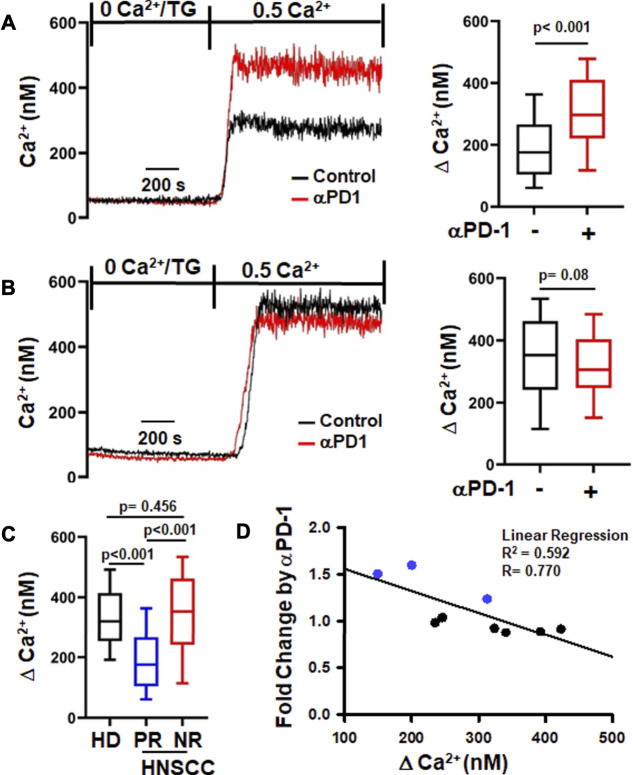
αPD-1 increases Ca^2+^ fluxes in PBTs of a subset of HNSCC patients. **(A,B)** Representative intracellular Ca^2+^ recordings in activated CD8^+^ PBTs of HNSCC patients are shown on the left side. Cells loaded with Fura-2 were perfused with thapsigargin (TG) in 0 mM Ca^2+^. Perfusion with 0.5 mM Ca^2+^ yields a rapid influx of Ca^2+^ (See *Material and Methods*). Two types of Ca^2+^ responses to αPD-1 were observed. A significant increase in Ca^2+^ after αPD-1 (10 μg/ml for 6 h) was defined as positive response, PR **(A, left)** while no change in Ca^2+^ response was defined as no-response, NR **(B, left)**. The subset of HNSCC patients (*n* = 3/9) showing positive Ca^2+^ response are reported in panel **(A, right)** while patients (*n* = 6/9) with no-response are shown in panel **(B, right)**. The corresponding single cell ∆Ca^2+^ values (peak minus baseline before peak) measured in the absence and presence of αPD-1 of PR and NR are shown in the right panels (*n* = 82–99 cells from 3 patients in PR and *n* = 140–149 cells from 6 patients in NR). **(C)** Comparison of ∆Ca^2+^ values of activated CD8^+^ PBTs from HDs *n* = 376 cells from nine donors and PR (*n* = 82 cells from 3 patients) and NR (*n* = 146 cells from 6 patients) HNSCC patients at baseline (before αPD-1). The ∆Ca^2+^ values in panels **(A,B,C)** are represented as box and whisker plots. The lower and upper bound of the box represent 25^th^ and 75^th^ percentiles respectively. Median values are shown as horizontal line in the box. The lower and upper error represents 10^th^ and 90^th^ percentile respectively. **(D)** Relationship between ∆Ca^2+^ values before αPD-1 treatment and fold increase in ∆Ca^2+^ after treatment with αPD-1 antibody in CD8^+^ PBTs of HNSCC patients (*n* = 9) are represented as correlation analysis. Individual patients from PR and NR group are marked in blue (PR) and black (NR). Data for panel **(A)** were analyzed using unpaired student’s *t*-test. Data for panel **(B)** were analyzed using Mann-Whitney rank-sum test. Data in panel C were analyzed using ANOVA (*p* < 0.001) followed by Dunn’s test and data in panel D were analyzed using linear regression.

Since K^+^ channels regulate T cell Ca^2+^ signaling and effector functions, we investigated the functional consequences of the increase in K^+^ channel activity in CD8^+^ PBTs of HNSCC patients induced by αPD-1 treatment. We measured the Ca^2+^ fluxing abilities of CD8^+^ PBTs from HNSCC patients using a TG-based protocol that allows us to assess Ca^2+^ fluxes dependent on ion channel activity while bypassing the TCR (Newton et al., 2020). Activated CD8^+^ PBTs from HNSCC patients were treated with αPD-1 for 6 h (same protocol as Figure 1) and Ca^2+^ fluxes were measured. We observed variable Ca^2+^ responses in these patients’ samples. Therefore, we defined the presence or absence of a response to αPD-1 based on a statistically significant increase in ΔCa^2+^ values before and after *in vitro* αPD-1 treatment in individual patients. In a subset of patients (*n* = 3/9), αPD-1 treatment increased the Ca^2+^ response of T cells (Figure 2A). We termed these patients as “positive-response” (PR) patients. Patients whose T cells did not show any increase in the Ca^2+^ response to αPD-1 treatment *in vitro* were defined as “no-response” (NR) patients (*n* = 6/9), (Figure 2B). αPD-1 treatment induced a 46% increase in ΔCa^2+^ in the PR group (Figure 2A). Interestingly, we observed that the ΔCa^2+^ at baseline (before αPD-1 treatment) of CD8^+^ PBTs from PR patients was 67% lower than NR patients; even lower than that of HDs CD8^+^ PBTs (Figure 2C). Indeed, we observed a significant negative correlation between the increase in ΔCa^2+^ (fold change for individual patient) induced by αPD-1 versus the baseline ΔCa^2+^ of all HNSCC patients (Figure 2D). We performed this type of analysis because we wanted to assess whether the mean baseline ΔCa^2+^ of a patient could be a predictor of his/her ability to respond to pembrolizumab. This indicates that HNSCC patients whose CD8^+^ PBTs have low baseline Ca^2+^ fluxing abilities were more likely to show a positive response to αPD-1 treatment. Overall, these results indicate that αPD-1 treatment has a rapid effect on ion channel-regulated Ca^2+^ fluxes of HNSCC CD8^+^ PBTs and point to baseline Ca^2+^ fluxing ability of T cells as a possible predictive marker of αPD-1 treatment response. However, questions still remain about the mechanisms by which αPD-1 antibody regulates K^+^ channel activity: is this effect the result of blocking PD-L1/PD-1 binding, and what are the downstream signaling pathways involved?

### The Effect of αPD-1 Treatment on K^+^ Channels is due to the Disruption of PD-L1/PD-1 Binding

**FIGURE 3:**
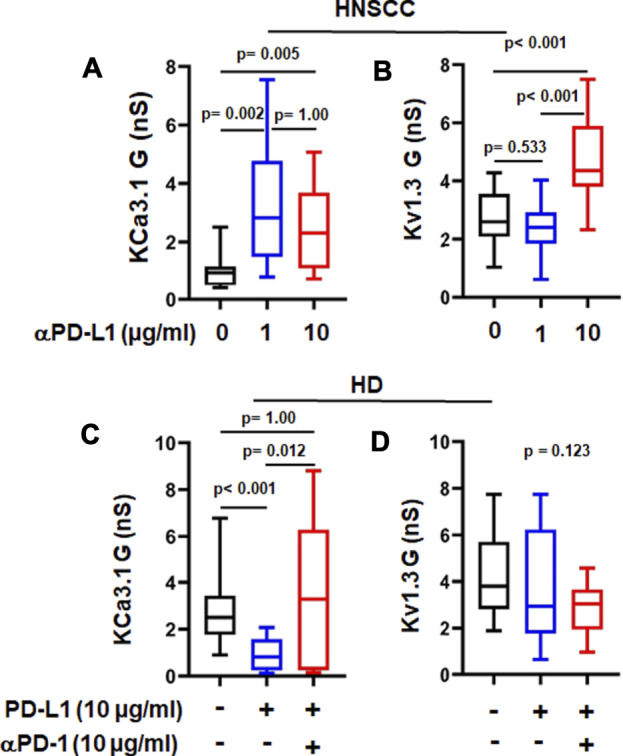
αPD-L1 treatment increases K^+^ channel activity in HNSCC patients. **(A)** KCa3.1 and **(B)** Kv1.3 conductance values **(G)** measured with and without the αPD-L1 antibody, atezolizumab (1 and 10 μg/ml for 6 h) in activated CD8^+^ PBTs of HNSCC patients. **(C)** KCa3.1 and **(D)** Kv1.3 G measured in presence of PD-L1 and αPD-1 antibody pembrolizumab in activated CD8^+^ PBTs of HDs. Activated cells were treated with plate-bound PD-L1 (PD-L1-Fc 10 μg/ml) +/- αPD-1 (untreated cells were used as a control) and activated for 72 h using PMA/Ionomycin. αPD-1 was added to treatment group for 6 h. Data in the lower and upper bound of the box represent 25^th^ and 75^th^ percentiles respectively. Median values are shown as horizontal lines. The lower and upper error bars represents 10^th^ and 90^th^ percentile respectively, *n* = 8–23 cells from 3 HNSCC patients, *n* = 30 cells from 6 HDs (control and PD-L1) and *n* = 15 cells from 3 HDs (PD-L1 + αPD-1). Five cells were recorded for each individual donor. Data in **(A,C,D)** were analyzed by ANOVA on ranks test (*p* < 0.001) followed by Dunn’s post hoc analysis. Data in **(B)** were analyzed by One way ANOVA (*p* < 0.001) followed by Holm-Sidak test.

We tested the corollary of our earlier observation with αPD-1 treatment hypothesizing that prevention of or breaking the interaction of PD-L1 with PD-1 is responsible for the improved K^+^ channel function. While we have no cancer cells in our *in vitro* setting, CD8^+^ PBTs of HDs and HNSCC patients can be a source of PD-L1 as both express it (Supplementary Figure S5). Others have shown that human CD3^+^ T cells express PD-L1 (Succaria et al., 2020). Therefore, we next tested the effect of an anti-PD-L1 antibody (αPD-L1), atezolizumab, on KCa3.1 and Kv1.3 channels in activated CD8^+^ PBTs of HNSCC patients reasoning that if the effect of pembrolizumab is due to the disruption of PD-L1/PD-1 binding, a similar effect should be produced by αPD-L1. Short-term treatment of HNSCC T cells with atezolizumab (1 and 10 μg/ml for 6 h) increased KCa3.1 function, while Kv1.3 function was increased only at the higher concentration (Figures 3A,B). These results suggest that engagement of PD-L1 by PD-1 may have negative effects on K^+^ channel function in HNSCC T cells and disruption of its binding to the cognate receptor restores the channel’s function. Therefore, we tested the effect of PD-L1 (plate-bound PD-L1-Fc; 10 μg/ml) on KCa3.1 and Kv1.3 in CD8^+^ PBTs of HDs. Cells were incubated with PD-L1 for 72 h followed by treatment with αPD-1 antibody (10 μg/ml) for 6 h. Our data showed that PD-L1 reduced KCa3.1 activity by 65.44%, but, at this concentration, had no effect on Kv1.3 (Figures 3C,D). A similar effect was observed after 5 days exposure to PD-L1 (Supplementary Figure S6). Furthermore, αPD-1 treatment reversed the effect of PD-L1 on KCa3.1 activity (Figure 3C). These results show that, in HDs CD8^+^ PBTs, KCa3.1 channels appear highly sensitive to PD-1 signaling, more than Kv1.3.

**FIGURE 4:**
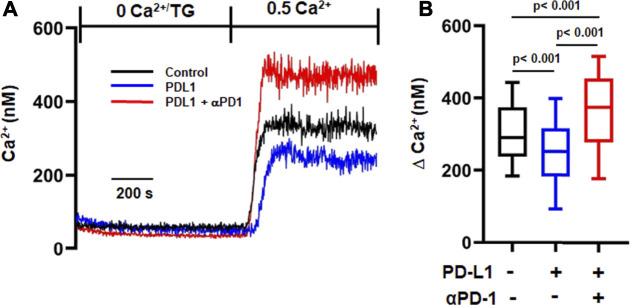
αPD-1 rescues the reduced Ca^2+^ influx induced by PDL-1-Fc in HDs. **(A)** Representative traces of intracellular Ca^2+^ response in activated CD8^+^ PBTs of HDs. Cells were loaded with Fura-2 and Ca^2+^ fluxes were elicited by TG/0 mM Ca^2+^ followed by 0.5 mM Ca^2+^ (See *Material and Methods*). Cells were incubated with plate bound PD-L1 (PD-L1-Fc, 10 μg/ml for 72 h) followed by treatment with αPD-1 (10 μg/ml for 6 h). **(B)** Summary of ∆Ca^2+^ values of activated CD8^+^ PBTs of HDs (n = 160–255 cells from nine HDs, 20–50 cells were recorded from a single HD, see *Methods*). The values in panel B are represented as box and whisker plots. The lower and upper bound of the box represent 25^th^ and 75^th^ percentiles respectively. Median values are shown as horizontal line. The lower and upper error bars represents 10^th^ and 90^th^ percentile respectively. Data were analyzed using ANOVA (*p* < 0.001) on ranks followed by Dunn’s *post hoc* analysis.

We then measured Ca^2+^ fluxes in activated CD8^+^ PBTs cells of HDs exposed to PD-L1 to assess the functional effect of PD-L1. Nine out of twelve samples from HDs showed response to PD-L1 and only the CD8^+^ PBTs samples from donors that showed this response were used in analysis. Cells exposed to PD-L1 showed a small (14%) decrease in ΔCa^2+^ as compared to control cells (Figure 4B). Treatment with pembrolizumab significantly increased ΔCa^2+^ by 39% in PD-L1 exposed cells, and by 25% in 9/12 HDs in the control untreated group (Figures 4A,B). It is noteworthy that treatment with pembrolizumab showed a similar increase of 45% in ΔCa^2+^ in HNSCC CD8^+^ PBTs (Figure 2A). These results indicate that PD-L1 reduced Ca^2+^ fluxing abilities of T cells in HDs thus supporting the notion that high exposure to PD-L1 may decrease the Ca^2+^ fluxing ability of HNSCC CD8^+^ PBTs like it occurs for TILs (Chimote et al., 2017). Furthermore, they suggest that KCa3.1 may be highly sensitive to PD-1 signaling, more than Kv1.3. We thus proceeded to investigate the mechanism through which PD-1 regulates KCa3.1 activity.

### PI3K Signaling and CaM Mediate PD-L1 inhibition of KCa3.1 Channels

**FIGURE 5:**
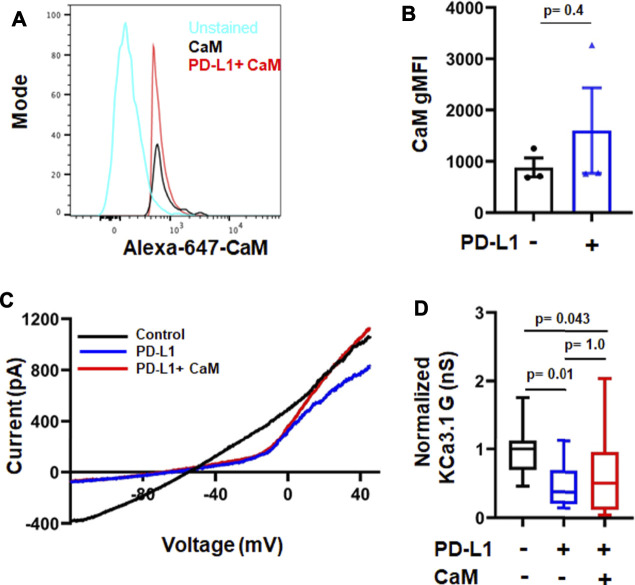
Short-time treatment with PD-L1 decreases KCa3.1 activity in a calmodulin-independent manner. **(A)** Flow cytometry histogram and geometric mean fluorescence intensity (gMFI) values **(B)** for CaM expression in activated CD8^+^ PBTs from HD donors (*n* = 3) in the absence and presence of PD-L1. **(C)** Representative recordings of KCa3.1 currents in activated CD8^+^ PBTs from HDs showing the effect of PD-L1 (PD-L1-Fc, 10 μg/ml) and CaM (50 µM). **(D)** Average normalized KCa3.1 conductance (**G**, nS) measured in the absence and presence of PD-L1, with and without CaM. All conductance values are normalized to average conductance value obtained from control recordings. Cells were pre-incubated with plate-bound PD-L1 (PD-L1-Fc,10 μg/ml, for 72 h) activated using anti-CD3/CD28 antibodies and treated with or without CaM (50 µM), that was delivered intracellularly *via* patch pipette during recordings (*n* = 15–18 cells per group from 3 HDs). The values in panel **(B)** are represented as bar graphs. Each symbol represent an individual HD. The values are represented as mean ± SEM. The values in panel **(D)** are represented as box and whisker plots. The lower and upper bound of the box represent 25^th^ and 75^th^ percentiles respectively. Median values are shown as horizontal line. The lower and upper error bars represents 10^th^ and 90^th^ percentile respectively. Data in panel **(B)** were analyzed by *t*-test and data in panel **(D)** were analyzed by ANOVA on ranks (*p* = 0.008) with Dunn’s *post hoc* analysis.

**FIGURE 6:**
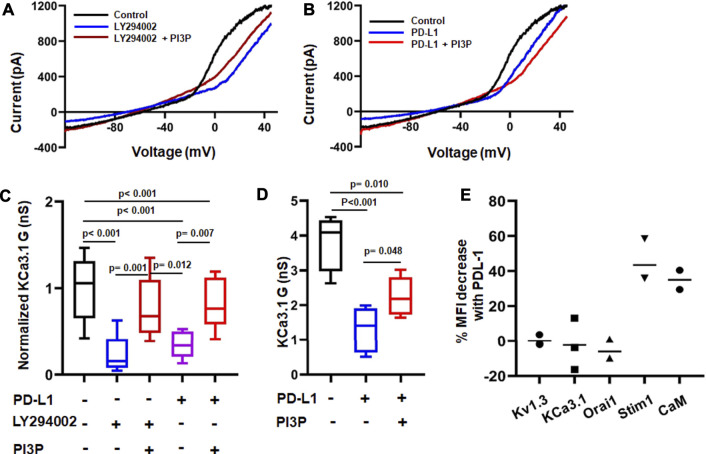
Differential time-dependent involvement of PI3K and calmodulin on PD-L1 mediated inhibition of KCa3.1 channels. **(A,B)** Representative recordings of KCa3.1 channels in activated CD8^+^ PBTs from HDs showing the effect of the PI3K inhibitor LY294002 (10 µM) +/− phosphatidylinositol-3 phosphatase (PI3P) (100 nM) **(A)** and PD-L1 (PD-L1-Fc, 10 μg/ml) +/− PI3P (100 nM) **(B). (C)** Summary of the pharmacological modulation of KCa3.1 channels byLY294002 and PI3P in the absence and presence of plate bound PD-L1 in activated CD8^+^ PBTs of HDs. Cells were activated using anti-CD3/CD28 antibodies for 72 h. Cells were perfused with LY294002 for 15 min followed by patch clamp recordings with and without PI3P, delivered intracellularly *via* patch pipette (*n* = eight to nine cells per group from 3 HDs). All KCa3.1 conductance **(G)** values are normalized to the average G of the control group (drug-free). **(D)** KCa3.1 G measured in absence or presence of PD-L1 in activated CD8^+^ PBTs of HDs. Cells were treated with plate-bound PD-L1 (PD-L1-Fc, 10 μg/ml) and activated using anti-CD3/CD28 antibodies for 120 h PI3P was delivered intracellularly *via* the patch pipette during the electrophysiological experiments (drug-free control). Cells were held at −70 mV, *n* = four to five cells per group from one HD. The values in panel **(C,D)** are represented as box and whiskers plot. The lower and upper bound of the box represent 25^th^ and 75^th^ percentiles respectively. Median values are shown as horizontal line. The lower and upper error bars represents 10^th^ and 90^th^ percentile respectively. **(E)** Percentage change in mean fluorescence intensity (MFI) of ion channels (Kv1.3, KCa3.1, Orai1, Stim1) and Calmodulin (CaM) measured using flow cytometry. Each dot represents an individual HD and the horizontal black line represents the mean value. Data in panel **(C)** were analyzed by One Way ANOVA (*p* < 0.001) followed by Holm-Sidak’s *post hoc* analysis. Data in **(D)** were analyzed by One way ANOVA followed by Holm-Sidak’s *post hoc* analysis.

Activation and function of KCa3.1 is controlled by a variety of molecules and phosphorylation events (Ohya and Kito, 2018). KCa3.1 function requires binding of the intracellular Ca^2+^ sensor calmodulin (CaM). We recently showed that CD8^+^ PBTs of HNSCC patients have reduced levels of CaM which diminished their KCa3.1 activity (Chimote et al., 2020). PD-L1 is available in the plasma of HNSCC patients both as soluble molecule or carried by exosomes, and the presence of PD-L1^+^ exosomes has been correlated with the increased immune suppressive state of these patients (Theodoraki et al., 2018). These findings raise the possibility that a reduction in CaM by PD-L1 mediates the suppression of KCa3.1 currents. Therefore, we proceeded to determine the signaling pathways involved in PD-L1/PD-1 signaling by exposing HD T cells to PD-L1 for 3 days (72 h; like in the experiments reported above) and 5 days. The latter resembles more the HNSCC patients’ setting where T cells are exposed to PD-L1, both in circulation and in the tumor, for a long time. Exposure to PD-L1 for 3 days did not reduce CaM expression (Figures 5A,B) and, consequently, intracellular supplementation of CaM did not rescue KCa3.1 inhibition (Figures 5C,D). We thus investigated whether the phosphoinositide 3-kinase (PI3K)—phosphatidylinositol-3 phosphatase (PI3P) signaling pathway was instead involved. In T lymphocytes, PI3K favors the production of PI3P from phosphatidylinositol (PI); PI3P activates the nucleoside diphosphate kinase B (NDPK-B) which, ultimately, increases KCa3.1 activity *via* histidine phosphorylation (Srivastava et al., 2005; Srivastava et al., 2006b). PD-1 signaling in T cells involves the blockade of PI3K and, consequently, the suppression of downstream effector functions (Patsoukis et al., 2013). We performed patch-clamp experiments to determine if the inhibition of KCa3.1 activity by PD-L1 can be mimicked by the PI3K inhibitor LY294002 and rescued by PI3P. Activated CD8^+^ PBTs from HDs were pretreated with LY294002 (10 μM, for 15 min) followed by measurement of KCa3.1 in presence and absence of PI3P (100 nM) delivered intracellularly *via* patch pipette (Figure 6A). LY294002, similar to PD-L1, reduced KCa3.1 currents while PI3P reversed this inhibition in both LY294002 and PD-L1 treated cells. (Figures 6A–C). These results provide the evidence of involvement of PI3K in the early (3 days) effect of PD-L1 on KCa3.1 activity. Longer exposure to PD-L1 revealed a diminished contribution of PI3K signaling and a role for CaM becomes evident (Figures 6D,E). Electrophysiological experiments showed that PI3P only partially restored the KCa3.1 activity of HDs CD8^+^ PBTs treated with PD-L1 for 5 days (Figure 6D). Flow-cytometry data showed that at this time point there was a significance 40% reduction in CaM expression and not KCa3.1. Interestingly, we observed also a selective reduction in Stim1 (the partner protein of Orai1 that forms CRAC channels) (Figure 6E). Overall, these findings support a role for PI3P signaling and CaM in mediating the effect of PD-L1/PD-1 interaction on KCa3.1 channels in CD8^+^ T cells.

## Discussion

Resistance to immunotherapy in cancer is attributed to a weak or blunted immune response. Engagement of the immune checkpoint receptor PD-1 by its ligand PD-L1 is part of the immune escape mechanism of cancer cells (Farhood et al., 2019). Herein, we provide evidence of a role for K^+^ channels as early regulators of PD-L1/PD-1 mediated suppression of cytotoxic T cells in HNSCC patients.

It is well established that blockade of PD-L1/PD-1 interaction increases T cell function and antitumor immunity (Alsaab et al., 2017). In this study, we report that disruption of PD-L1/PD-1 binding by αPD-1 and αPD-L1 antibodies, rapidly (within 6 h) increased KCa3.1 and Kv1.3 activities in CD8^+^ PBTs of HNSCC patients**.** Studies in murine models and human subjects with solid malignancies show that αPD-1 treatment improves T cell activation as indicated by the increased expression of activation markers (Peng et al., 2012; Ascierto et al., 2019). Herein, we showed that short treatment with αPD-1 antibody (pembrolizumab) was not sufficient to change the expression of the T cell activation marker CD69, and yet produced changes in the activity of K^+^ channels. These data suggest that activation of K^+^ channels may be among the earliest changes produced by interruption of PD-1 signaling in CD8^+^ PBTs of HNSCC patients. We also showed that αPD-1 treatment affected exclusively K^+^ channels as it did not alter CRAC channels. Furthermore, the effect of αPD-1 antibody on K^+^ channels could not be ascribed to changes in channel protein expression or activation/exhaustion state of T cells as markers of T cell activation and exhaustion and ion channel proteins were all unchanged. This is in agreement with what we observed in T cells from HNSCC patients treated with pembrolizumab (Newton et al., 2020).

A well-established functional consequence of increased KCa3.1 and Kv1.3 activities in T cells is an increase in Ca^2+^ fluxing abilities that is necessary for cytokine production and cytotoxicity (Feske et al., 2012). In the present study, we measured TCR-independent Ca^2+^ fluxes to assess the consequences of the positive effect of αPD-1 treatment on K^+^ channel activity. Indeed, we observed that pembrolizumab produces a rapid increase in Ca^2+^ fluxing abilities of CD8^+^ PBTs in a subset of HNSCC patients. The *in vitro* positive Ca^2+^ response to pembrolizumab was observed in 33% of patients. This response is in line with the objective response reported in a recent clinical trial (KEYNOTE-048) in HNSCC patients (Burtness et al., 2019; Kok, 2020). We also observed that a low baseline Ca^2+^ fluxing ability of CD8^+^ PBTs from HNSCC patients predicts the response to pembrolizumab. It is possible that HNSCC patients whose T cells display lower Ca^2+^ fluxing abilities *in vitro* had a higher exposure to circulating PD-L1 (either soluble or *via* tumor-derived exosomes) *in vivo*, and they will ultimately benefit the most from αPD-L1 and αPD-1 blockade. An *in vitro* study showed that PD-L1 decreases TCR dependent Ca^2+^ fluxes in a dose dependent manner in human T cells overexpressing the PD-1 receptor (Wei et al., 2013). In agreement with this study, we reported here that PD-L1 inhibits ion channel-dependent Ca^2+^ fluxes in CD8^+^ T cells. Furthermore, multiple studies have shown that a high PD-L1 expression in cancer cells correlate with a good response to αPD-1/αPDL-1 blocking antibodies (Evrard et al., 2020).

Our data showed that while KCa3.1 and Kv1.3 channels mediate the response to αPD-1 and αPD-L1 antibodies, there may be a difference in sensitivity. Indeed, a higher concentration of αPD-L1 was necessary to unleash Kv1.3 activity in HNSCC T cells. Furthermore, the concentration of PD-L1 activating antibodies (PD-L1-Fc) we used (10 μg/ml) suppressed KCa3.1, but not Kv1.3, currents in CD8^+^ PBTs of HDs, and, in this setting, αPD-1 antibody increased KCa3.1, but not Kv1.3, conductance. It is possible that inhibition of Kv1.3 requires higher concentrations of PDL-1 and/or a longer exposure. Our earlier observations *in vivo* in HNSCC patients treated with pembrolizumab showed increases KCa3.1, but not Kv1.3, channels activity in CD8^+^ PBTs shortly after treatment (Newton et al., 2020). The effect of PD-1 blockade on Kv1.3 is only evident a long time after pembrolizumab administration. However, it is also possible that higher levels of PD-L1 may be needed for Kv1.3 inhibition. This could explain what we observed untreated HNSCC patients (Chimote et al., 2018; Newton et al., 2020). We reported low KCa3.1 activity in CD8^+^ PBTs, which is further reduced in TILs (Chimote et al., 2018; Newton et al., 2020). However, while we did not observe any reduction in Kv1.3 activity in HNSCC circulating T cells, there was a profound suppression of this channel activity/expression in TILs, particularly those in close proximity with cancer cells, (Chimote et al., 2018; Newton et al., 2020) where the levels of PD-L1 are higher than in circulation. Secreted PD-L1 is detected in the plasma of HNSCC patients contributing to reduced T cell function (Zhou et al., 2020). While we showed that CD8^+^ PBTs express PD-L1, we do not know the PD-L1 levels in our cell cultures and how they compare with those of PD-L1-Fc used in our experiments. The presence of T cell generated PD-L1 (endogenous PD-L1) explains the response to αPD-1 and αPD-L1 antibodies without pre-treatment with PD-L1-Fc (Figures 1, 3). It is very likely that PD-L1-Fc is in addition to the endogenous PD-L1. This explains why αPD-1 brings the Ca^2+^ response of PD-L1-Fc treated cells to levels higher than “untreated” cells that are exposed to the endogenous PD-L1 (Figure 4B).

The high sensitivity of KCa3.1 to PD-L1 may be explained by the mechanisms by which PD-1 regulates this channel which imply a fast kinase-mediated signaling event. We observed that the response of KCa3.1 channels to PD-1 ligation is mediated by PI3K-PI3P signaling and that this is the earliest pathway engaged. CaM downregulation follows in time. In T cells, activation of TCR signaling cascade leads to activation of PI3K/Akt [phosphatidylinositol 3-kinase (PI3K) and Akt/Protein Kinase B)], while ligation of PD-1 results in a decrease in TCR proximal signaling through inhibition of PI3K activity (Seidel et al., 2018). PI3K has also been implicated in KCa3.1 regulation (Ohya and Kito, 2018). KCa3.1 channels have a histidine residue (H358) in the carboxy-terminus whose phosphorylation is regulated by PI3K, PI3P and NDPK-B kinase (Srivastava et al., 2006b; Srivastava et al., 2009; Srivastava et al., 2016). KCa3.1 channels in healthy human CD4^+^ T cells are inhibited by the non-selective PI3K blocker LY294002, while PI3P (whose formation from PI is facilitated by PI3K) reverses this inhibition (Srivastava et al., 2006a). NDPK-B (the enzyme mediating PI3P effect on KCa3.1) knock-down decreases KCa3.1 without affecting the activity of Kv1.3 (Srivastava et al., 2006b). Similarly, in this study we showed that PD-L1-Fc at the concentration we used only inhibited KCa3.1, that this effect was reproduced by LY294002, and reversed by PI3P. While the PI3K/PI3P signaling pathway is the dominant regulatory mechanism early upon PD-1 ligation, a reduction in CaM expression contributes to reduced KCa3.1 activity later on. CaM serves as binding partner and intracellular Ca^2+^ sensor for KCa3.1 allowing the activation of this Ca^2+^-dependent channel (Cahalan and Chandy, 2009; Sforna et al., 2018). We have recently reported that membrane CaM levels are downregulated in activated CD8^+^ T cells of HNSCC patients (Chimote et al., 2020). In this study, the long-term effect of PD-1 ligation in healthy CD8^+^ T cells is a decrease in CaM expression. CaM downregulation is not contributing to KCa3.1 suppression at earlier time points as indicated by the lack of effect of intracellular supplementation of CaM on KCa3.1 channels in PBTs of HDs treated for 72 h with PD-L1-Fc. At this stage, it is not clear if there is a correlation between PI3K and CaM. However, it is possible that the decrease in Ca^2+^ fluxing abilities that follows the reduction in KCa3.1 by PI3K inhibition may lead to reduced CaM. It has been reported that CaM gene expression is regulated by Ca^2+^ (Bosch et al., 1994).

Overall, our *in vitro* studies showed that αPD-1 induces an increase in Kv1.3, KCa3.1 and Ca^2+^ fluxing that ultimately will lead to improved anti-tumor response. Ca^2+^ signaling in fact regulates multiple effector functions of T cells, including the production of cytokines like IL-2 and IFN-γ, and cytotoxicity (Feske et al., 2003). These are highly desirable anti-tumor functions. In agreement with the studies presented here, we have recently reported that *in vivo* administration of αPD-1 blockade followed by surgical resection increases Ca^2+^ fluxing abilities of CD8^+^ T cells by increasing the KCa3.1 and Kv1.3 channel function in head and neck cancer patients (Newton et al., 2020). Furthermore, a recent report on combination of immune checkpoint inhibitors, anti-PD-1 and anti-LAG-3 antibodies, showed significant increase in peak Ca^2+^ levels of T cells resulting in increased cytotoxicity (Sullivan et al., 2020).

In conclusion, the data presented in this manuscript highlight the crucial role of K^+^ channels as early modulators of PD-1 signaling and point to them as therapeutic targets in restoring anti-tumor immunity in HNSCC patients. This study paves the way for further investigations about new therapeutic strategies in cancer that incorporate K^+^ channel activators along with immune checkpoint inhibitors.

## Data Availability

The original contributions presented in the study are included in the article/Supplementary Material, further inquiries can be directed to the corresponding author/s.

## References

[B1] Alsaab H. O. Sau S. Alzhrani R. Tatiparti K. Bhise K. Kashaw S. K. (2017). PD-1 and PD-L1 Checkpoint Signaling Inhibition for Cancer Immunotherapy: Mechanism, Combinations, and Clinical Outcome. Front. Pharmacol. 8, 561. 10.3389/fphar.2017.00561 28878676 PMC5572324

[B2] Ascierto P. A. Bifulco C. Buonaguro L. Emens L. A. Ferris R. L. Fox B. A. (2019). Perspectives in Immunotherapy: Meeting Report from the “Immunotherapy Bridge 2018” (28-29 November, 2018, Naples, Italy). J. Immunother. Cancer 7 (1), 332. 10.1186/s40425-019-0798-3 31783779 PMC6884742

[B3] Bauml J. M. Aggarwal C. Cohen R. B. (2019). Immunotherapy for Head and Neck Cancer: where Are We Now and where Are We Going? Ann. Transl. Med. 7 (Suppl. 3), S75. 10.21037/atm.2019.03.58 31576284 PMC6685871

[B4] Bosch M. López-Girona A. Bachs O. Agell N. (1994). Protein Kinase C Regulates Calmodulin Expression in NRK Cells Activated to Proliferate from Quiescence. Cell Calcium 16 (6), 446–454. 10.1016/0143-4160(94)90074-4 7712538

[B5] Burtness B. Harrington K. J. Greil R. Soulières D. Tahara M. de Castro G. Jr. (2019). Pembrolizumab Alone or with Chemotherapy versus Cetuximab with Chemotherapy for Recurrent or Metastatic Squamous Cell Carcinoma of the Head and Neck (KEYNOTE-048): a Randomised, Open-Label, Phase 3 Study. Lancet 394 (10212), 1915–1928. 10.1016/s0140-6736(19)32591-7 31679945

[B6] Cahalan M. D. Chandy K. G. (2009). The Functional Network of Ion Channels in T Lymphocytes. Immunol. Rev. 231 (1), 59–87. 10.1111/j.1600-065X.2009.00816.x 19754890 PMC3133616

[B7] Chapon M. Randriamampita C. Maubec E. Badoual C. Fouquet S. Wang S. F. (2011). Progressive Upregulation of PD-1 in Primary and Metastatic Melanomas Associated with Blunted TCR Signaling in Infiltrating T Lymphocytes. J. Invest. Dermatol. 131 (6), 1300–1307. 10.1038/jid.2011.30 21346771

[B8] Chimote A. A. Balajthy A. Arnold M. J. Newton H. S. Hajdu P. Qualtieri J. (2018). A Defect in KCa3.1 Channel Activity Limits the Ability of CD8+ T Cells from Cancer Patients to Infiltrate an Adenosine-Rich Microenvironment. Sci. Signal. 11 (527), eaaq1616. 10.1126/scisignal.aaq1616 29692361 PMC6006512

[B9] Chimote A. A. Gawali V. S. Newton H. S. Wise-Draper T. M. Conforti L. (2020). A Compartmentalized Reduction in Membrane-Proximal Calmodulin Reduces the Immune Surveillance Capabilities of CD8+ T Cells in Head and Neck Cancer. Front. Pharmacol. 11, 143. 10.3389/fphar.2020.00143 32184726 PMC7059094

[B10] Chimote A. A. Hajdu P. Kottyan L. C. Harley J. B. Yun Y. Conforti L. (2016). Nanovesicle-targeted Kv1.3 Knockdown in Memory T Cells Suppresses CD40L Expression and Memory Phenotype. J. Autoimmun. 69, 86–93. 10.1016/j.jaut.2016.03.004 26994905 PMC4830342

[B11] Chimote A. A. Hajdu P. Kucher V. Boiko N. Kuras Z. Szilagyi O. (2013). Selective Inhibition of KCa3.1 Channels Mediates Adenosine Regulation of the Motility of Human T Cells. J. Immunol. 191 (12), 6273–6280. 10.4049/jimmunol.1300702 24227782 PMC4415878

[B12] Chimote A. A. Hajdu P. Sfyris A. M. Gleich B. N. Wise-Draper T. Casper K. A. (2017). Kv1.3 Channels Mark Functionally Competent CD8+ Tumor-Infiltrating Lymphocytes in Head and Neck Cancer. Cancer Res. 77 (1), 53–61. 10.1158/0008-5472.CAN-16-2372 27815390 PMC5215046

[B13] Chow L. Q. M. (2020). Head and Neck Cancer. N. Engl. J. Med. 382 (1), 60–72. 10.1056/NEJMra1715715 31893516

[B14] Evrard D. Hourseau M. Couvelard A. Paradis V. Gauthier H. Raymond E. (2020). PD-L1 Expression in the Microenvironment and the Response to Checkpoint Inhibitors in Head and Neck Squamous Cell Carcinoma. Oncoimmunology 9 (1), 1844403. 10.1080/2162402X.2020.1844403 33299655 PMC7714503

[B15] Farhood B. Najafi M. Mortezaee K. (2019). CD8+ Cytotoxic T Lymphocytes in Cancer Immunotherapy: A Review. J. Cel. Physiol. 234 (6), 8509–8521. 10.1002/jcp.27782 30520029

[B16] Feske S. Gwack Y. Prakriya M. Srikanth S. Puppel S. H. Tanasa B. (2006). A Mutation in Orai1 Causes Immune Deficiency by Abrogating CRAC Channel Function. Nature 441 (7090), 179–185. 10.1038/nature04702 16582901

[B17] Feske S. Okamura H. Hogan P. G. Rao A. (2003). Ca2+/calcineurin Signalling in Cells of the Immune System. Biochem. Biophys. Res. Commun. 311 (4), 1117–1132. 10.1016/j.bbrc.2003.09.174 14623298

[B18] Feske S. Skolnik E. Y. Prakriya M. (2012). Ion Channels and Transporters in Lymphocyte Function and Immunity. Nat. Rev. Immunol. 12 (7), 532–547. 10.1038/nri3233 22699833 PMC3670817

[B19] Feske S. Wulff H. Skolnik E. Y. (2015). Ion Channels in Innate and Adaptive Immunity. Annu. Rev. Immunol. 33, 291–353. 10.1146/annurev-immunol-032414-112212 25861976 PMC4822408

[B20] Kok V. C. (2020). Current Understanding of the Mechanisms Underlying Immune Evasion from PD-1/pd-L1 Immune Checkpoint Blockade in Head and Neck Cancer. Front. Oncol. 10, 268. 10.3389/fonc.2020.00268 32185135 PMC7058818

[B21] Newton H. S. Gawali V. S. Chimote A. A. Lehn M. A. Palackdharry S. M. Hinrichs B. H. (2020). PD1 Blockade Enhances K+ Channel Activity, Ca2+ Signaling, and Migratory Ability in Cytotoxic T Lymphocytes of Patients with Head and Neck Cancer. J. Immunother. Cancer 8 (2), e000844. 10.1136/jitc-2020-000844 33060146 PMC7566435

[B22] Ohya S. Kito H. (2018). Ca2+-Activated K+ Channel KCa3.1 as a Therapeutic Target for Immune Disorders. Biol. Pharm. Bull. 41 (8), 1158–1163. 10.1248/bpb.b18-00078 30068864

[B23] Patsoukis N. Li L. Sari D. Petkova V. Boussiotis V. A. (2013). PD-1 Increases PTEN Phosphatase Activity while Decreasing PTEN Protein Stability by Inhibiting Casein Kinase 2. Mol. Cel. Biol. 33 (16), 3091–3098. 10.1128/MCB.00319-13 PMC3753920 23732914

[B24] Peng W. Liu C. Xu C. Lou Y. Chen J. Yang Y. (2012). PD-1 Blockade Enhances T-Cell Migration to Tumors by Elevating IFN-γ Inducible Chemokines. Cancer Res. 72 (20), 5209–5218. 10.1158/0008-5472.Can-12-1187 22915761 PMC3476734

[B25] Robbins J. R. Lee S. M. Filipovich A. H. Szigligeti P. Neumeier L. Petrovic M. (2005). Hypoxia Modulates Early Events in T Cell Receptor-Mediated Activation in Human T Lymphocytes via Kv1.3 Channels. J. Physiol. 564 (Pt 1), 131–143. 10.1113/jphysiol.2004.081893 15677684 PMC1456048

[B26] Seidel J. A. Otsuka A. Kabashima K. (2018). Anti-PD-1 and Anti-CTLA-4 Therapies in Cancer: Mechanisms of Action, Efficacy, and Limitations. Front. Oncol. 8, 86. 10.3389/fonc.2018.00086 29644214 PMC5883082

[B27] Sforna L. Megaro A. Pessia M. Franciolini F. Catacuzzeno L. (2018). Structure, Gating and Basic Functions of the Ca2+-Activated K Channel of Intermediate Conductance. Curr. Neuropharmacol. 16 (5), 608–617. 10.2174/1570159x15666170830122402 28875832 PMC5997868

[B28] Sharma P. Hu-Lieskovan S. Wargo J. A. Ribas A. (2017). Primary, Adaptive, and Acquired Resistance to Cancer Immunotherapy. Cell 168 (4), 707–723. 10.1016/j.cell.2017.01.017 28187290 PMC5391692

[B29] Srivastava S. Choudhury P. Li Z. Liu G. Nadkarni V. Ko K. (2006a). Phosphatidylinositol 3-phosphate Indirectly Activates KCa3.1 via 14 Amino Acids in the Carboxy Terminus of KCa3.1. Mol. Biol. Cel. 17 (1), 146–154. 10.1091/mbc.e05-08-0763 PMC1345654 16251351

[B30] Srivastava S. Di L. Zhdanova O. Li Z. Vardhana S. Wan Q. (2009). The Class II Phosphatidylinositol 3 Kinase C2beta Is Required for the Activation of the K+ Channel KCa3.1 and CD4 T-Cells. Mol. Biol. Cel. 20 (17), 3783–3791. 10.1091/mbc.e09-05-0390 PMC2735477 19587117

[B31] Srivastava S. Li Z. Ko K. Choudhury P. Albaqumi M. Johnson A. K. (2006b). Histidine Phosphorylation of the Potassium Channel KCa3.1 by Nucleoside Diphosphate Kinase B Is Required for Activation of KCa3.1 and CD4 T Cells. Mol. Cel. 24 (5), 665–675. 10.1016/j.molcel.2006.11.012 17157250

[B32] Srivastava S. Li Z. Lin L. Liu G. Ko K. Coetzee W. A. (2005). The Phosphatidylinositol 3-phosphate Phosphatase Myotubularin- Related Protein 6 (MTMR6) Is a Negative Regulator of the Ca2+-Activated K+ Channel KCa3.1. Mol. Cel. Biol. 25 (9), 3630–3638. 10.1128/mcb.25.9.3630-3638.2005 PMC1084293 15831468

[B33] Srivastava S. Panda S. Li Z. Fuhs S. R. Hunter T. Thiele D. J. (2016). Histidine Phosphorylation Relieves Copper Inhibition in the Mammalian Potassium Channel KCa3.1. Elife 5, e16093. 10.7554/eLife.16093 27542194 PMC5005030

[B34] Succaria F. Kvistborg P. Stein J. E. Engle E. L. McMiller T. L. Rooper L. M. (2020). Characterization of the Tumor Immune Microenvironment in Human Papillomavirus-Positive and -negative Head and Neck Squamous Cell Carcinomas. Cancer Immunol. Immunother. 70, 1227–1237. 10.1007/s00262-020-02747-w 33125511 PMC8188514

[B35] Sullivan M. R. Ugolini G. S. Sarkar S. Kang W. Smith E. C. McKenney S. (2020). Quantifying the Efficacy of Checkpoint Inhibitors on CD8+ Cytotoxic T Cells for Immunotherapeutic Applications via Single-Cell Interaction. Cell Death Dis. 11 (11), 979. 10.1038/s41419-020-03173-7 33188167 PMC7666200

[B36] Sung H. Ferlay J. Siegel R. L. Laversanne M. Soerjomataram I. Jemal A. (2021). Global Cancer Statistics 2020: GLOBOCAN Estimates of Incidence and Mortality Worldwide for 36 Cancers in 185 Countries. CA A. Cancer J. Clin. 71, 209–249. 10.3322/caac.21660 33538338

[B37] Theodoraki M. N. Yerneni S. S. Hoffmann T. K. Gooding W. E. Whiteside T. L. (2018). Clinical Significance of PD-L1+ Exosomes in Plasma of Head and Neck Cancer Patients. Clin. Cancer Res. 24 (4), 896–905. 10.1158/1078-0432.Ccr-17-2664 29233903 PMC6126905

[B38] Vaeth M. Yang J. Yamashita M. Zee I. Eckstein M. Knosp C. (2017). ORAI2 Modulates Store-Operated Calcium Entry and T Cell-Mediated Immunity. Nat. Commun. 8, 14714. 10.1038/ncomms14714 28294127 PMC5355949

[B39] Wei F. Zhong S. Ma Z. Kong H. Medvec A. Ahmed R. (2013). Strength of PD-1 Signaling Differentially Affects T-Cell Effector Functions. Proc. Natl. Acad. Sci. U S A. 110 (27), E2480–E2489. 10.1073/pnas.1305394110 23610399 PMC3703988

[B40] Zhou J. Mahoney K. M. Giobbie-Hurder A. Zhao F. Lee S. Liao X. (2017). Soluble PD-L1 as a Biomarker in Malignant Melanoma Treated with Checkpoint Blockade. Cancer Immunol. Res. 5 (6), 480–492. 10.1158/2326-6066.Cir-16-0329 28522460 PMC5642913

[B41] Zhou K. Guo S. Li F. Sun Q. Liang G. (2020). Exosomal PD-L1: New Insights into Tumor Immune Escape Mechanisms and Therapeutic Strategies. Front. Cel. Dev. Biol. 8, 569219. 10.3389/fcell.2020.569219 PMC7593554 33178688

