# Supplementary material for: Immune Checkpoint Inhibitors Regulate K+ Channel Activity in Cytotoxic T Lymphocytes of Head and Neck Cancer Patients
Source: Front Pharmacol. 2021 Aug 27;12:742862. doi: 10.3389/fphar.2021.742862 (PMC8429813; doi:10.3389/fphar.2021.742862)
Supplement: Supplementary file 1 [file DataSheet1.DOCX]

**Supplementary Figures**

**
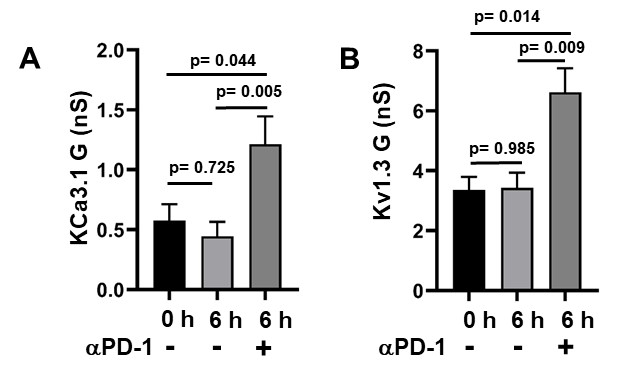
**

**Figure S1.**

**Increase in KCa3.1 and Kv1.3 channel activity by αPD-1 is independent of time.** **A-B** CD8^+^ PBTs from HNSCC patients were activated for 72-96 h followed by treatment with αPD-1 (10 µg/ml) for 6 h. **A)** KCa3.1 and **(B)** Kv1.3 conductance, G, values measured in untreated activated CD8^+^ PBTs at 0 h (72-96 hr after the initial activation) and after an additional 6 hours of activation with and without αPD-1 (10 µg/ml) treatment. This latter time corresponds to the time when cells treated with αPD-1 (10 µg/ml) were tested. For panels A & B bars represent mean ± SEM of n= 15 cells from 3 patients. Data were analyzed by ANOVA on ranks test (p=0.004) with Tukey’s test.

**
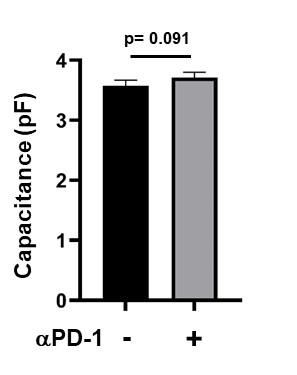
**

**Figure S2.**

**Effect of αPD-1 treatment on cell capacitance of HNSCC CD8^+^ cells.** Capacitance values (pF), measured in cells evaluated for KCa3.1 and Kv1.3 currents in absence and presence of αPD-1 (10 µg/ml, for 6 h) in CD8^+^ PBT cells of HNSCC patients. Data are represented in a bar graph as mean ± SEM (n= 68 cells without αPD-1 and n= 56 cells with αPD-1 treatment from 14 HNSCC patients; same experiments as Figure S1). Data were analyzed by Mann-Whitney rank sum test.

**
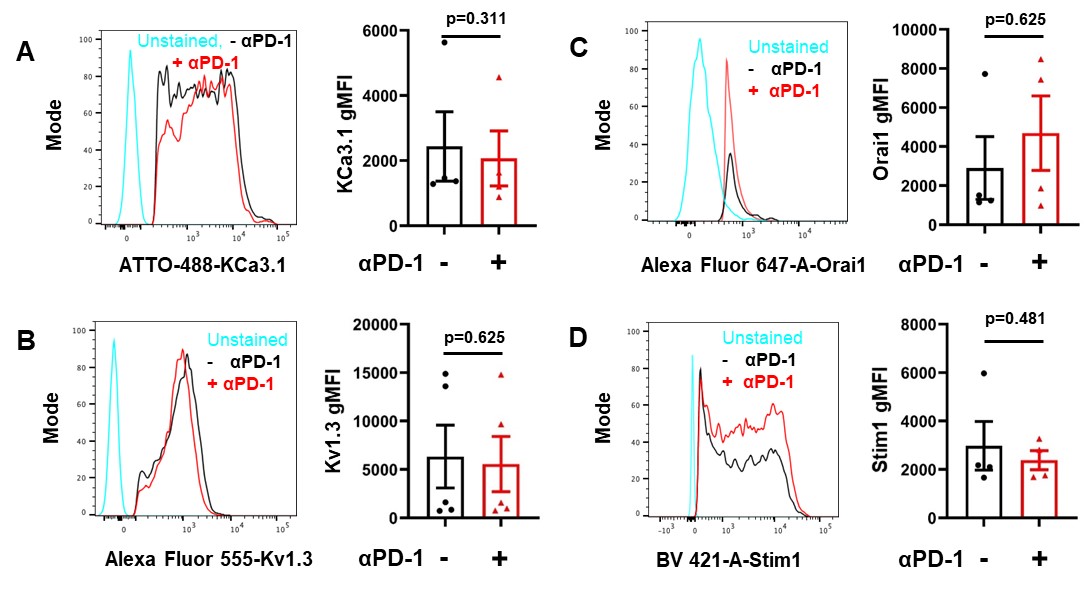
Figure S3.**

**αPD-1 treatment did not change ion channel expression in PBTs of HNSCC patients.** Flow cytometry histogram and geometric mean fluorescence intensity (gMFI) values are shown for KCa3.1 **(A)**, Kv1.3 **(B)**, Orai1 **(C)**, and Stim1 **(D)** expression in the absence and presence of αPD-1 in PBTs of HNSCC patients**.** gMFI values are represented in bar graphs. Each data point represents an individual patient. (n=4-5 patients per group). Bars represents mean ± SEM. Data in panel B and C were analyzed by signed rank test and data in panel A and D were analyzed by paired Student’s t-test.

**
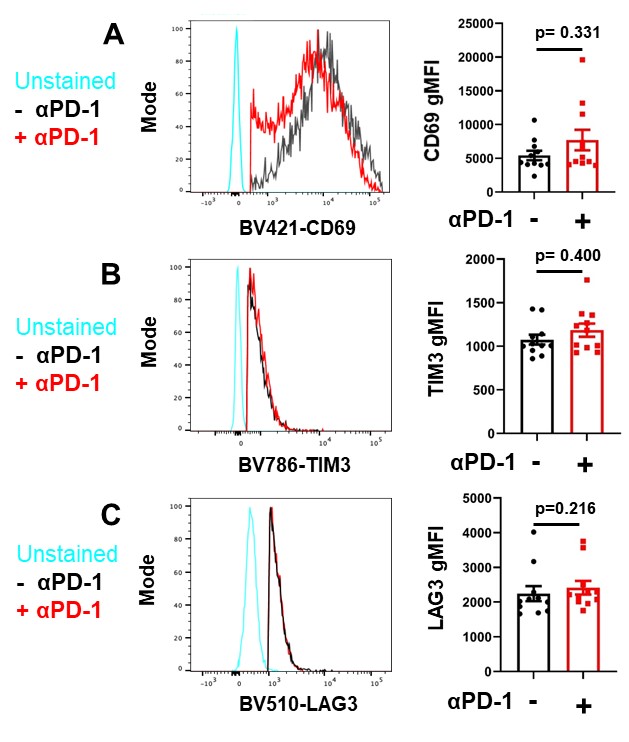
**

**Figure S4.**

**Expression of T cell activation and exhaustion markers are unaffected by αPD-1 treatment.** Representative flow cytometry overlay histograms are shown in left and geometric mean fluorescence intensity (gMFI) are shown at right. gMFI values are shown for T cell activation marker CD69 **(A)**, T cell exhaustion markers TIM3 **(B)**, and LAG-3 **(C)** expression on CD8^+^ PBTs from HNSCC patients with or without αPD-1 treatment. Right panels in A-C are shown as bar graphs with data point represents an individual patient with mean ± SEM for each group (n= 11 patients). Expression of all above markers were measured in a live cell population identified by exclusion from Zombie Aqua live/dead staining. Data were analyzed by Mann-Whitney rank sum test.


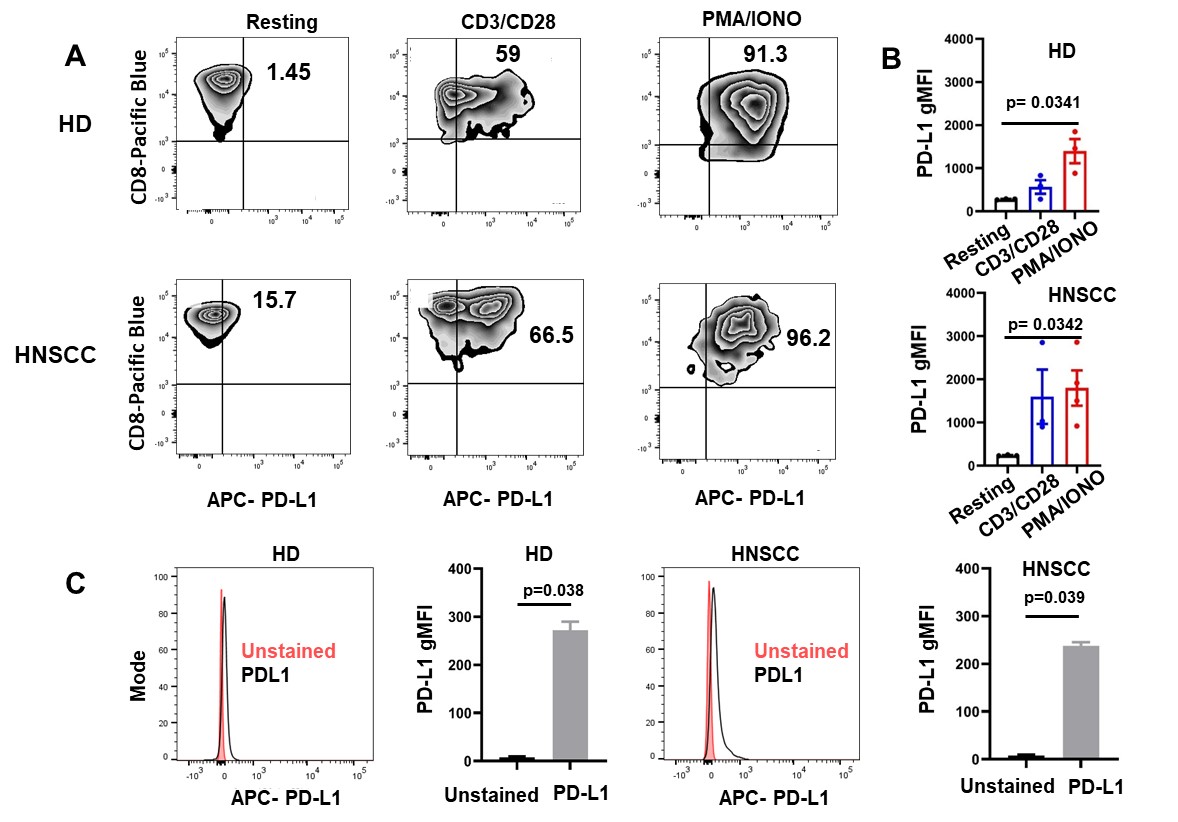


**Figure S5.**

**PD-L1 expression in T cells of HNSCC patients and healthy donors. A)** Representative flow cytometry plots in resting and activated CD8^+^ PBTs from HD and HNSCC patients showing PD-L1 expression. Cells were stimulated using CD3 and CD28 antibodies and PMA/Ionomycin as described in the *Materials and Methods*. Live cell population was identified by exclusion from Zombie Aqua live/dead stain and gated on PD-L1^+^ T cells. Percentages of PD-L1^+^ CD8^+^ PBTs are shown in the upper right quadrant. **(B)** Geometric mean fluorescence intensity (gMFI) values for PD-L1 expression in resting and activated CD8^+^ PBTs of HD (n=3) and HNSCC (n=3) patients. (**C)** Representative flow cytometry overlay histograms showing PD-L1 expression in resting CD8^+^ PBTs from HD (left) and HNSCC (right) patient. Quantification of PD-L1 expression is shown by gMFI values for PD-L1 expression in resting CD8^+^ T cells of HD (n=3) and HNSCC (n=3) patients in bar graphs. Bars represent Mean ± SEM. Data in panel B were analyzed by one way ANOVA. Data in panel C were analyzed by Two-tailed, student’s t-test


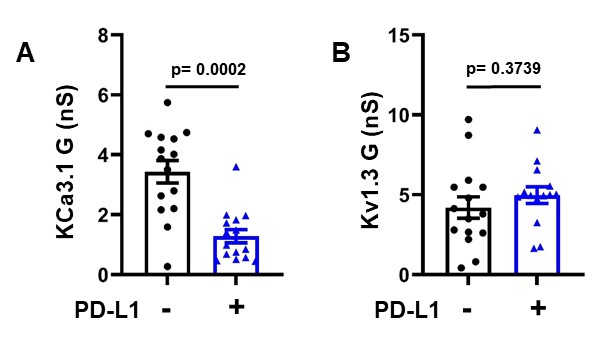


**Figure S6.**

**Prolonged exposure to PD-L1 decreases KCa3.1 activity.** **A)** KCa3.1 and (**B**) Kv1.3 conductance, G values measured in activated CD8^+^ PBTs of HDs in absence and presence of PD-L1 (PD-L1-Fc 10 µg/ml) for 5 days. Cells were activated using CD3 and CD28 antibodies for 5 days in medium supplemented with 20 IU/mL IL-2. Bar graphs in A and B represent mean ± SEM. Each symbol represents an individual cell (n= 15 cells from 3 HDs). Data were analyzed by paired t-test.

**Supplementary Table S1.**

**Clinical and pathological characteristics of HNSCC patients**. Clinical staging, tumor location and gender information for each HNSCC patient are shown. Pathological feature HPV status (p16) is included in the table.

|  | Patient ID | Patient Sex | Head and Neck Disease Site | Clinical Stage | p16 status |
| --- | --- | --- | --- | --- | --- |
| 1 | HNC-93 | Female | Oral Cavity | T2N0M0 | Negative |
| 2 | HNC-97 | Male | Unknown | Unknown | Negative |
| 3 | HNC-109 | Female | Oral | T1N1 | Negative |
| 4 | HNC-114 | Male | Larynx | T4N0 | Unknown |
| 5 | HNC-115 | Male | Larynx | T3N2 | Unknown |
| 6 | HNC-116 | Male | Larynx | T4N1 | Unknown |
| 7 | HNC-118 | Male | Oral Cavity | T1N2 | Negative |
| 8 | HNC-122 | Female | Oral Cavity | T1N2 | Negative |
| 9 | HNC-124 | Female | Larynx | T1N0 | Positive |
| 10 | HNC-135 | Male | Larynx | T3N1 | Unknown |
| 11 | HNC-159 | Male | Oropharynx | T1N2 | Positive |
| 12 | HNC-161 | Female | Larynx | T2N3 | Unknown |
| 13 | HNC-166 | Male | Oropharynx | T2N0 | Negative |
| 14 | HNC-170 | Female | Oropharynx | T2N2 | Positive |
| 15 | HNC-172 | Male | Oropharynx | T1N1 | Positive |
| 16 | HNC-173 | Male | Oral Cavity | T2N0 | Unknown |
| 17 | HNC-174 | Male | Oral Cavity | T4N3 | Negative |
| 18 | HNC-176 | Female | Oral Cavity | T3N2 | Negative |
| 19 | HNC-177 | Female | Oropharynx | T3N2 | Positive |
| 20 | HNC-188 | Male | Oral Cavity | T2N1 | Unknown |
| 21 | HNC-189 | Male | Oral Cavity | T4N1 | Negative |
| 22 | HNC-190 | Male | Oral Cavity | T4N0 | Negative |
| 23 | HNC-193 | Male | Larynx | T4N2 | Positive |
| 24 | HNC-194 | Male | Oropharynx | T2N1 | Negative |
| 25 | HNC-213 | Male | Oropharynx | T4N2 | Negative |
| 26 | HNC-214 | Female | Oropharynx | T3N2 | Positive |
| 27 | HNC-215 | Male | Oropharynx | T2N0 | Positive |
| 28 | HNC-216 | Male | Oropharynx | T1N1 | Positive |
| 29 | HNC-228 | Male | Oropharynx | T2N1 | Positive |
| 30 | HNC-234 | Male | Oropharynx | T2N3 | Negative |
| 31 | HNC-235 | Male | Oropharynx | T2N1 | Positive |
| 32 | HNC-237 | Male | Oropharynx | T1N1 | Positive |
